# Vertical root fracture resistance of simulated immature permanent teeth filled with MTA using different vehicles

**DOI:** 10.4317/jced.53121

**Published:** 2017-02-01

**Authors:** Hacer Aksel, Sevinc Askerbeyli-Örs, Derya Deniz-Sungur

**Affiliations:** 1 DDS, PhD, Department of Endodontics, Faculty of Dentistry, Hacettepe University, Ankara, Turkey; 2 DDS, Department of Endodontics, Faculty of Dentistry, Hacettepe University, Ankara, Turkey

## Abstract

**Background:**

The aim of the study is to evaluate the resistance vertical root fracture (VRF) of mineral trioxide aggregate (MTA) filled-immature permanent roots by using three different vehicles.

**Material and Methods:**

Forty-extracted human single-rooted mandibular premolars were selected and the root length was standardized to the length of 9 mm. For simulation of immature tooth apices, peeso reamers were introduced into the root canals and the prepared roots were assigned into three experimental groups according the used vehicle (distilled water-DW, prophylene glycol-PG, chlorhexidine-CHX) and control group (n=10). To simulate a periodontal membrane, the apical 7 mm of all roots was covered with wax to obtain a 0.2- to 0.3-mm-thick layer before embedding the roots into acrylic cylinders. A vertical force was applied (1mm/min) using a universal testing machine and the maximum load (F-max) that fracture occurred and the fracture mode (splint or comminuted) was recorded. Data were presented as mean and standard deviations. Statistical analysis was performed using Kruskal-Wallis, Mann-Whitney U Test was used for multiple comparisons.

**Results:**

There were significant differences between fracture strength of experimental groups with that of control group (*p*<0.05). However, no statistically significant differences were found amongst the fracture strength values of the experimental groups (*p*>0.05). In all groups, split fracture was the most common fracture mode.

**Conclusions:**

MTA increases resistance of immature permanent teeth to VRF. Based on the results of this study, it can be concluded that mixing MTA with CHX or PG as the vehicle do not alter VRF resistance of simulated immature permanent roots.

** Key words:**Immature teeth, MTA, vehicle, vertical root fracture.

## Introduction

The treatment of necrotic immature permanent teeth has always been a clinical challenge for several reasons. It is difficult to achieve an appropriate apical seal with an open apex by using conventional root canal obturation methods. Apexification and revascularization have been used for the treatment of these cases (1). Revascularization has been shown to be an effective treatment alternative for thickening and lengthening of the weak root canals (2-4) but may not be successful in every case (5,6). In these conditions, obturation of the full-length root canals with a bicompatible and insoluble material which completely fill the irregularities through the immature root canals.

Mineral trioxide aggregate (MTA) has been used as a biocompatible obturation material for necrotic immature permanent teeth to achieve an appropriate apical seal (7,8). MTA has been mixed with different vehicles instead of distilled water (DW) such as chlorhexidine (CHX) and propylene glycol (PG). CHX is a cationic bisguanide with a strong antibacterial activity against a wide range of gram positive and gram negative organisms, yeast, facultative anaerobes and aerobes (9). A mixture of white MTA and CHX showed significantly more antimicrobial activity than white MTA and DW (10). In another study, mixing 2% CHX with MTA powder was showed to have a significant increase in the antibacterial effect of white MTA and gray MTA against *E. faecalis* (11).

Another vehicle to improve the handling and physical properties of MTA is PG (12). It is a viscous alcoholic compound approved by FDA. In the literature, its activity against common endodontic pathogens and improved ability to penetrate into dentin, compared to DW has been shown (13). Other studies showed that mixing MTA with PG improves its sealing ability, flowability, pH, and calcium ion release (14).

Although these vehicles affect the physical properties of MTA, the effect of these vehicles on fracture resistance of immature root canal wall has not yet been evaluated. For this reason, the aim of this study is to reveal the effects of mixing MTA with different vehicles on the vertical root fracture resistance of immature permanent teeth.

## Material and Methods

-Specimen Preparation

All experiments were performed under a protocol approved by the Human Subjects Ethical Review Committee of Hacettepe University.

Forty extracted human single-rooted mandibular premolars, with straight root canals were used. The teeth devoid of caries, fracture or cracks upon examination under a stereoscopic microscope at ×32 magnifications were selected. Furthermore, only teeth with similar mesio-distal and buccolingual root dimensions were included (±8%). The crowns were removed using a water-cooled diamond disc at the cemento-enemal junction (CEJ), and 9 mm-roots in length were obtained by also resecting the root end. The root canals were prepared using ProTaper NiTi instruments (Dentsply Maillefer, Ballaigues, Switzerland). Each root canal was irrigated with 2 mL of distilled water between successive instruments. To simulate teeth with immature apices, peeso reamers between #1 and #6 (1.70 mm) were used to enlarge the root apex by allowing them to protrude 1 mm beyond the root apex. At the end of instrumentation, each root canal was irrigated with 5 mL of 17% ethylenediaminetetraacetic acid (EDTA) and 10 mL of 2.5% NaOCl to remove the smear layer and then rinsed with 5 mL of distilled water. The root canals were dried with paper points, and the external surfaces were dried by air blast. All specimens were stored at 100% relative humidity during the experimental period.

-Experimental Procedure

The prepared specimens were divided into three experimental groups according to the vehicle used and a control group (n = 10); group 1- MTA was mixed with DW, group 2-MTA was mixed with PG with the ratio of 80% DW and 20% PG, group 3- MTA was mixed with 2% CHX and control group- filled with gutta percha and sealer. (AH Plus, Dentsply De Trey, Konstanz, Germany) by lateral compaction technique.

**Figure 1:**
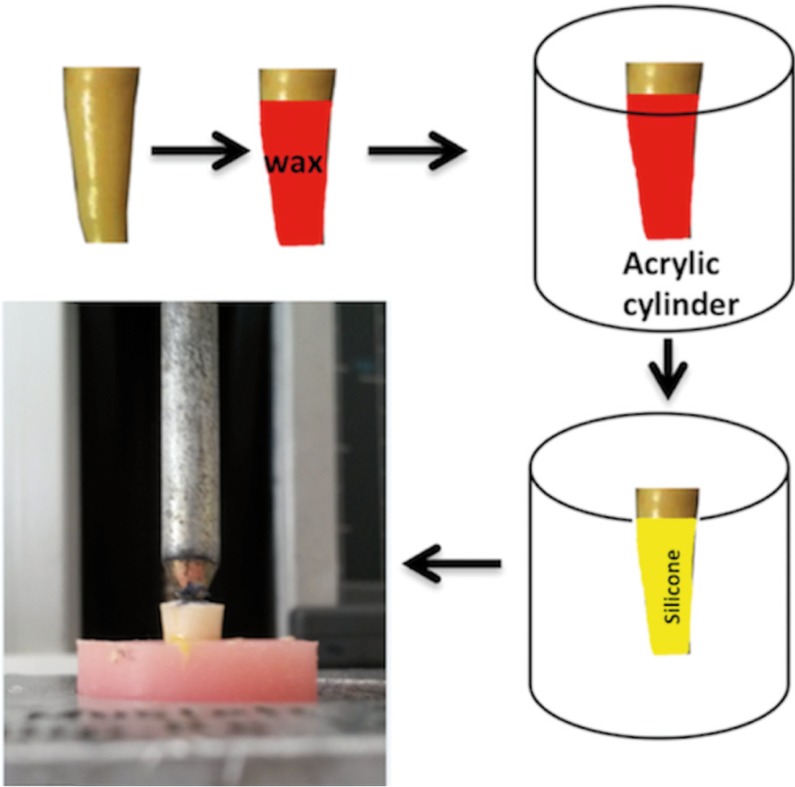
Simulation of the periodontal ligament-Roots dipped in molten wax. Wax boiled out and replaced with light-body silicone impression material. Root accurately embedded in acrylic socket.

 All specimens were kept in saline at 37°C for 2 weeks and saline was renewed on a weekly basis. The apical 7 mm of roots was covered with approximately 0.2 - 0.3 mm thickness of wax for periodontal membrane simulation before embedding the roots into acrylic cylinders (Fig. 1). After setting of the acrylic resin, the roots were removed and the acrylic sockets were washed out with boiling water to ensure total removal of the wax layer. The acrylic sockets were dried and filled with light body silicone impression material. Immediately, each root was positioned in its acrylic socket. Each specimen was mounted in an universal testing machine (Instron, Instron Corp, Canton, MA) and then subjected to the vertical load with an application tip at a crosshead speed of 1 mm/min. The maximum load that fracture occurred (F-max) and the fracture mode were recorded. The data were statistically analyzed using Kruskal-Wallis test, and Mann-Whitney-U test was used for pair-wise comparisons with SPSS software version 21.00, (SPSS Inc., Chicago, IL, USA). The level of statistical significance was set at *P* < 0.05.

## Results

**Figure 2:**
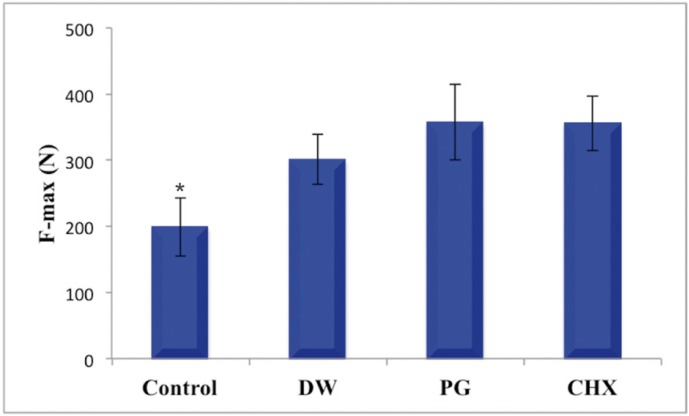
Bar chart illustrating the means and standard deviations of F-max at fracture within each group and (*) indicates statistical difference.

The mean F-max values of the groups were as follows: 198.76 ± 43.63 N for control group, 300.90 ± 37.67 N for DW, 357.71± 57.26 N for PG, 355.59 ± 40.85 N for CHX group (Fig. 2). There were significant differences between fracture strength of experimental groups with that of control group (*P*<0.05). However, no significant differences were observed amongst the experimental groups (*P*>0.05).

**Figure 3:**
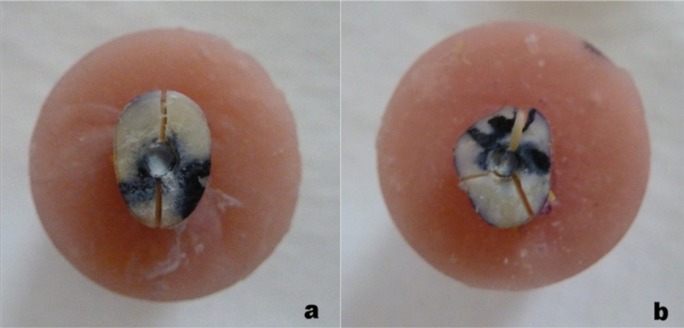
Types of fracture: a) split b) comminuted fracture.

Two fracture modes were detected (Fig. 3); a split vertical fracture that extended along the long axis of the root and a comminuted fracture that shattered the root into fragments. The most common fracture mode was the split root fracture in all groups with the incidence of 90% for control group, 70% for DW and 60% for both PG and CHX groups.

## Discussion

In the present study, an *in vitro* model stimulates immature permanent teeth to evaluate the vertical fracture resistance after filled with MTA mixed with different vehicles. Mesio-distal and bucco-lingual diameters of the roots were measured at the level of CEJ and the teeth were distributed into groups according to the similar diameters.

Previous studies have used bovine teeth, sheep teeth or mature human teeth to mimic immature tooth model for mechanical testing (15-18). Based on the previous studies, the experimental model consisted of mature teeth instrumented with #6 peeaso reamer (1.7 mm diameter) to simulate immature root development (18,19).

Endodontically treated teeth are at high risk of root fracture (20). Although there has been variation among the sites of vertical root fracture in various studies, the highest prevalence occurs in premolars (21). For this reason, mandibular premolars were selected and immature root formation was simulated to measure resistance to vertical root fracture of immature permanent premolars in this study.

In *in vitro* fracture resistance test, the root embedment material should reproduce bone that can absorb masticatory loads and resist the compressive and tangential forces. Periodontal ligament simulation prevents stress concentration in one particular region, and transfers the stresses produced by load application all along the root surface. Acrylic blocks and silicone impression material were used to simulate the bone and periodontal ligament, respectively (15). There were 2 mm gap between the mounting material and CEJ. This gap is considered to provide physiological distance found clinically between the bone crest and CEJ (22).

MTA-treated teeth were reported to show an initial decrease in fracture strength, however after 2 months, the process was reversed and the strength was increased after 1 year (23). For this reason, it is important to prevent this initial decrease in the fracture resistance of MTA-treated teeth. In this study, we consider to test different vehicles to reveal the effect on the fracture resistance of MTA filled immature roots. No significant difference was observed in the fracture resistance of simulated immature teeth after filling the root canals with MTA mixed distilled water, CHX or PG.

Mixing MTA with sterile water was found to have higher compressive strength than mixing with CHX (11). This can be related to the unsetting of MTA mixed with CHX after 72 hours. Since a previous study showed that MTA mixed with 2% CHX gel did not set even after 7 days (24). In our study, the specimens were stored 14 days to ensure complete set of filling materials.

PG is a successful vehicle for some root canal sealers to facilitate their placement into the canal and improve antibacterial activity (25,26). This is due to the fact that the pH and calcium release increase with the addition of PG into MTA (14). Furthermore, Holland *et al.* (27) evaluated dogs’ periapical tissue response after filling the root canals with MTA mixed with either PG or DW and showed that PG facilitates the placement of MTA into the canal with no influence on its biocompatibility. Mixing MTA with PG also increases its push-out bond strength to dentin, then it can be proposed that the resistance to vertical root fracture increases (28). The findings of this study showed that the greatest fracture strength values were achieved with PG group. PG was used with the ratio of 80% DW - 20% PG in this study, since this ratio has been recommended to improve physical and chemical properties of MTA (14).

Another finding was the fracture mode within each group. In all groups, split fracture that extended along the entire root length splitting it into 2 parts was the most commonly encountered fracture type. The incidence of the split fracture was higher in control group than MTA group regardless of the used vehicle. Comminuted fracture that occurs at higher forces was observed in MTA filled roots indicating higher fracture resistance of MTA filled roots.

Within the limitation of this *in vitro* study, mixing with PG or CHX instead of DW has no influence on the vertical root fracture resistance of MTA in immature permanent teeth. However, MTA increased the resistance compared to the control group. MTA can be mixed with PG with the ratio of 80% DW-20% PG or CHX without altering vertical root fracture resistance of immature permanent teeth.
